# A guide to selecting high-performing antibodies for PLC-gamma-2 for use in Western Blot, immunoprecipitation and immunofluorescence

**DOI:** 10.12688/f1000research.146156.1

**Published:** 2024-01-18

**Authors:** Vera Ruíz Moleón, Maryam Fotouhi, Charles Alende, Riham Ayoubi, Logan M. Bedford, Kathleen Southern, Timothy I. Richardson, Carl Laflamme, NeuroSGC/YCharOS/EDDU collaborative group, ABIF consortium

**Affiliations:** 1 Department of Neurology and Neurosurgery, Structural Genomics Consortium, The Montreal Neurological Institute, McGill University, Montreal, Québec, H3A 2B4, Canada; 2 Department of Pharmacology and Toxicology, Indiana University School of Medicine, Indianapolis, Indiana, 46202, USA

**Keywords:** Uniprot ID P16885, PLCG2, PLC-gamma-2, PLC-γ2, Phosphatidylinositol-specific phospholipase C gamma 2, antibody characterization, antibody validation, Western Blot, immunoprecipitation, immunofluorescence

## Abstract

Phosphatidylinositol-specific phospholipase C gamma 2 (PLC-gamma-2) is an enzyme that regulates the function of immune cells. PLC-gamma-2 has been implicated in neurodegenerative and autoimmune disorders, yet investigation of this protein has been limited by a lack of independently characterized antibodies. Here we have characterized eleven PLC-gamma-2 commercial antibodies for use in Western Blot, immunoprecipitation, and immunofluorescence using a standardized experimental protocol based on comparing read-outs in knockout cell lines and isogenic parental controls. These studies are part of a larger, collaborative initiative seeking to address antibody reproducibility issues by characterizing commercially available antibodies for human proteins and publishing the results openly as a resource for the scientific community. While use of antibodies and protocols vary between laboratories, we encourage readers to use this report as a guide to select the most appropriate antibodies for their specific needs.

## Introduction

Phosphatidylinositol-specific phospholipase C gamma 2 (PLC-gamma-2) is expressed primarily in immune cells. ^1^ It belongs to a family of phospholipases that facilitate the cleavage of phosphatidylinositol 4,5-bisphosphate into inositol 1,4,5-triphosphate (IP3) and diacylglycerol (DAG). ^2^ DAG activates protein kinase C ^3^ while IP3 diffuses through the cytosol to the endoplasmic reticulum where it stimulates the release of calcium stores. ^4^ By regulating these second messengers, PLC-gamma-2 regulates cellular functions such as migration, adhesion, calcium signaling, and phagocytosis. ^5^^,^^6^

In the absence of activation, PLC-gamma-2 exists in an autoinhibited state in the cytosol. ^7^^,^^8^ In order for PLC-gamma-2 to be recruited to the plasma membrane, phosphorylation is required. This phosphorylation induces domain reorganization of the autoinhibitory core and exposes the catalytic domain to its substrate, IP3, at the inner membrane surface. ^9^

The particular kinases and scaffolding proteins that mediate the activation of PLC-gamma-2 are dependent on the receptor being activated. Unlike its related family members, PLC-gamma-2 is activated downstream of receptor tyrosine kinases rather than G protein coupled receptors. ^2^

PLC-gamma-2 has been implicated in a variety of diseases. Gain-of function point mutations cause autoinflammatory disease. ^10^^,^^11^ Patients with rheumatoid arthritis have upregulated PLC-gamma-2 expression in peripheral blood mononuclear cells. ^12^ PLC-gamma-2 signaling can drive leukemogenesis in cases of Ibrutinib refractory chronic lymphocytic leukemia. ^13^^,^^14^ Additionally, PLC-gamma-2 is involved in solid cancers through Wnt signaling. ^15^ Finally, whole-exome microarray data identified a rare hypermorphic variant (P522R) associated with decreased risk of Alzheimer’s Disease (AD). ^16^^–^^20^ Conversely, a loss-of-function variant (M28L) is associated with increased risk. ^21^ These risk modifying variants suggest that PLC-gamma-2 may be a potential target for the treatment of AD and related dementia. ^22^^,^^23^ Studies aimed at elucidating the mechanistic role of PLC-gamma-2 in signaling pathways relevant to neurodegenerative processes involved in these diseases would benefit greatly from the availability of well characterized, high-quality antibodies.

This research is part of a broader collaborative initiative in which academics, funders and commercial antibody manufacturers are working together to address antibody reproducibility issues by characterizing commercial antibodies for human proteins using standardized protocols, and openly sharing the data ^24^^–^^26^ Here, we evaluated the performance of eleven commercially-available antibodies for PLC-gamma-2 in Western Blot, immunoprecipitation, and immunofluorescence using a knockout based approach. This article serves as a valuable guide to help researchers select high-quality antibodies for their specific needs, facilitating the biochemical and cellular assessment of PLC-gamma-2 properties and function.

## Results and discussion

Our standard protocol involves comparing readouts from wild-type (WT) and knockout (KO) cells. ^27^^,^^28^ The first step is to identify a cell line(s) that expresses sufficient endogenous levels of a given protein to generate a measurable signal. To this end, we examined the DepMap transcriptomics database to identify all cell lines that express the target at levels greater than 2.5 log _2_ (transcripts per million “TPM” + 1), which we have found to be a suitable cut-off (Cancer Dependency Map Portal, RRID:SCR_017655). Commercially available THP-1 cells expressed the PLC-gamma-2 transcript at RNA levels above the average range of cancer cells analyzed. Parental and *PLCG2* KO THP-1 cells were obtained from Abcam ( Table 1).

**Table 1.** Summary of the cell lines used.

Institution	Catalog number	RRID (Cellosaurus)	Cell line	Genotype
Abcam	ab271147	CVCL_0006	THP-1	WT
Abcam	ab308482	-	THP-1	*PLCG2* KO

**Figure 1.**
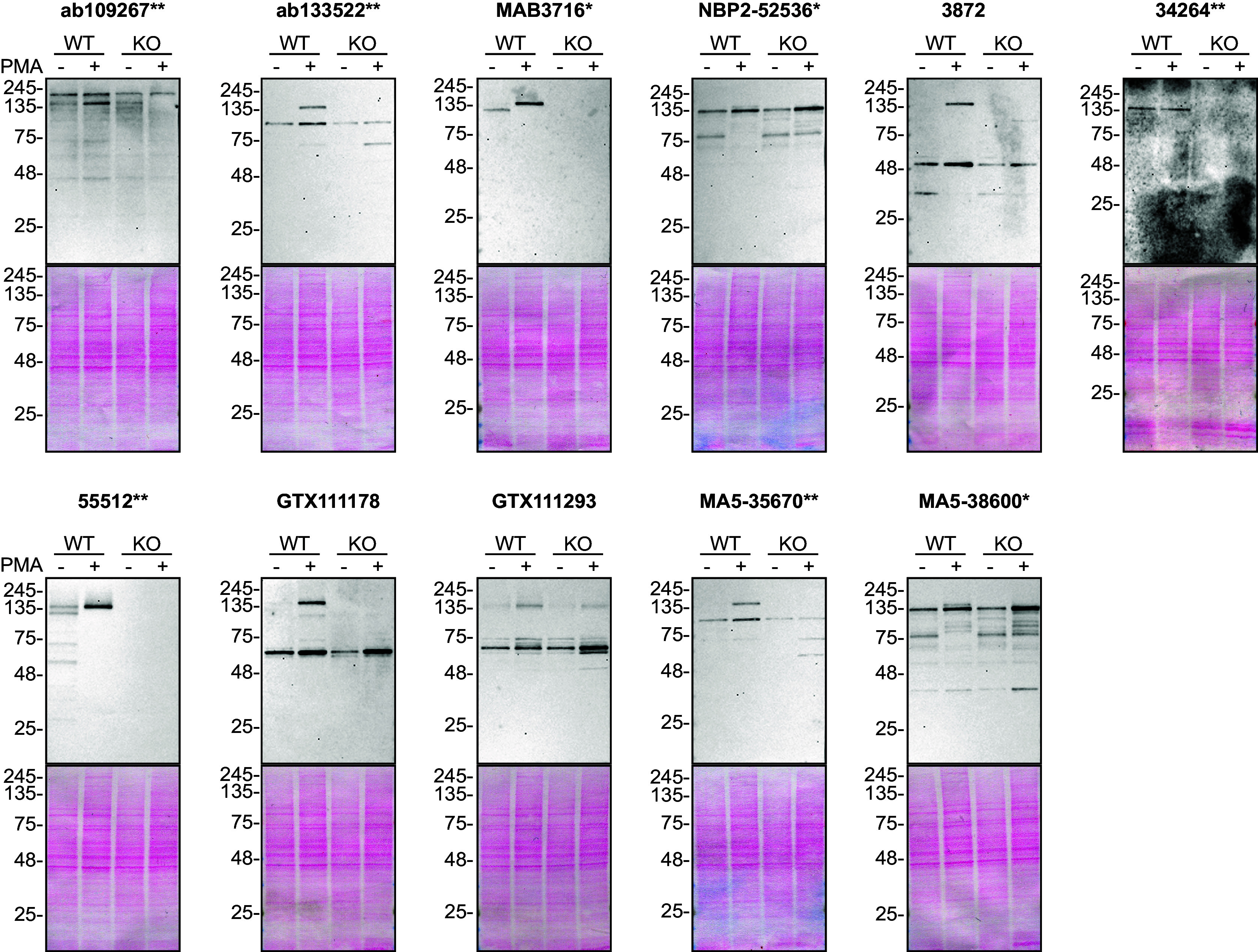
PLC-gamma-2 antibody screening by Western Blot. Lysates of THP-1 (WT and *PLCG2* KO) either treated (+) or not treated (-) with PMA were prepared and 10 μg of protein were processed for Western Blot with the indicated PLC-gamma-2 antibodies. The Ponceau stained transfers of each blot are presented to show equal loading of WT and KO lysates and protein transfer efficiency from the acrylamide gels to the nitrocellulose membrane. Antibody dilutions were chosen according to the recommendations of the antibody supplier. All antibodies were tested at a dilution of 1/500 except antibody 34264** was tested at 1/200. Predicted band size: 148 kDa. *Monoclonal antibody, **Recombinant antibody.

For Western Blot analyses, we resolved proteins from WT and *PLCG2* KO cell extracts. Both WT and KO cell lines were treated with and without phorbol 12-myristate 13-acetate (PMA) and then probed side-by-side with all antibodies in parallel ( Figure 1).

**Figure 2.**
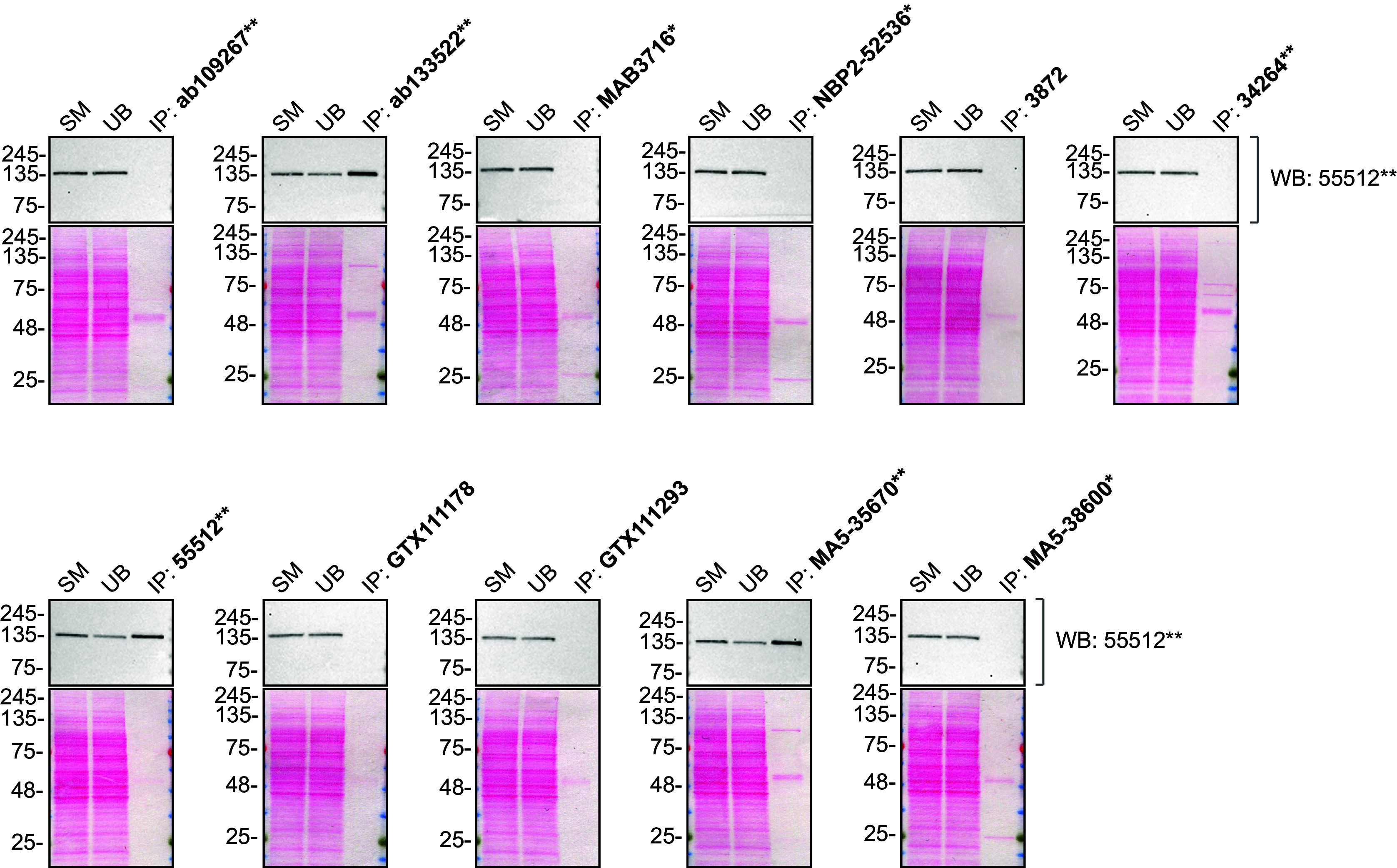
PLC-gamma-2 antibody screening by immunoprecipitation. THP-1 lysates were prepared, and immunoprecipitation was performed using 2.0 μg of the indicated PLC-gamma-2 antibodies pre-coupled to Dynabeads protein G or protein A. Samples were washed and processed for Western Blot with the indicated PLC-gamma-2 antibody. For Western Blot, 55512** was used at 1/500. The Ponceau stained transfers of each blot are shown. SM=4% starting material; UB=4% unbound fraction; IP=immunoprecipitate. *Monoclonal antibody, **Recombinant antibody.

As per our standard protocol, we next used the antibodies to immunoprecipitate PLC-gamma-2 from THP-1 WT and KO cell extracts. The performance of each antibody was evaluated by detecting the PLC-gamma-2 protein in extracts, in the immunodepleted extracts and in the immunoprecipitates ( Figure 2).

**Figure 3.**
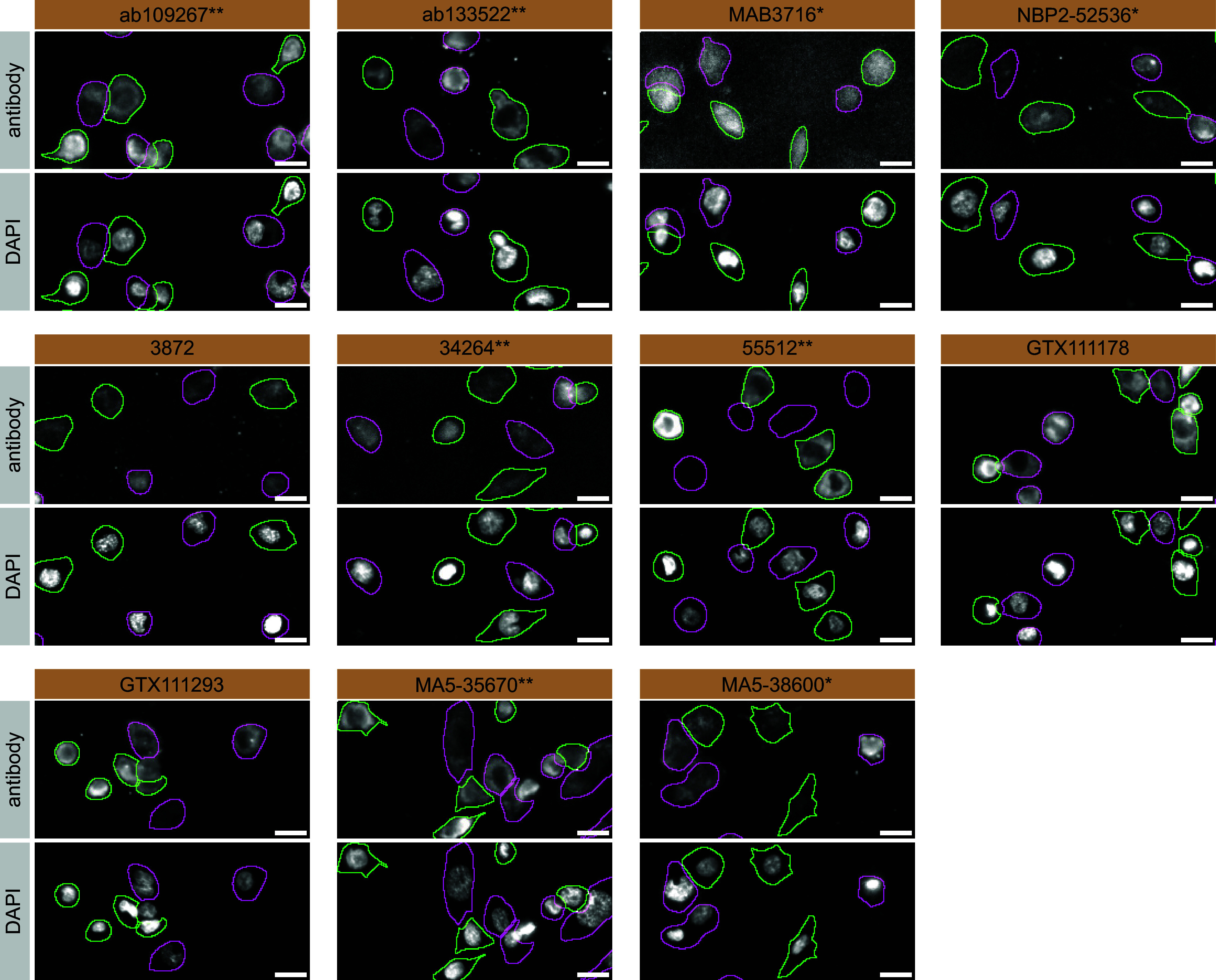
PLC-gamma-2 antibody screening by immunofluorescence. THP-1 WT and *PLCG2* KO cells were labelled with a green or a far-red fluorescent dye, respectively. WT and KO cells were mixed and plated to a 1:1 ratio in a 96-well plate with optically clear flat-bottom. Cells were stained with the indicated PLC-gamma-2 antibodies and with the corresponding Alexa-fluor 555 coupled secondary antibody including DAPI. Acquisition of the blue (nucleus-DAPI), green (WT), red (antibody staining) and far-red (KO) channels was performed. Representative images of the merged blue and red (grayscale) channels are shown. WT and KO cells are outlined with green and magenta dashed line, respectively. Antibodies were tested at 1.0 μg/mL. Antibody dilution used: ab109267** at 1/1300, ab133522** at 1/600, MAB3716* at 1/500, NBP2-52536* at 1/1000, 3872 at 1/1000, 34264** at 1/102, 55512** at 1/50, GTX111178 at 1/100, GTX111293 at 1/700, MA5-35670** at 1/700 and MA5-38600* at 1/1000. Bars = 10 μm. *Monoclonal antibody, **Recombinant antibody.

For immunofluorescence antibodies were screened using a mosaic strategy, as per our standardized protocol. First, THP-1 WT and *PLCG2* KO cell lines were labelled with different coloured fluorescent dyes, in order to distinguish the two cell lines, and the eleven PLC-gamma-2 antibodies were evaluated. Cells were imaged in the same field of view to reduce staining, imaging and image analysis bias ( Figure 3). Quantification of immunofluorescence intensity in hundreds of WT and KO cells was performed for each antibody tested. The images presented in Figure 3 are representative of the results of this analysis.

In summary, we have screened eleven PLC-gamma-2 commercial antibodies by Western Blot, immunoprecipitation and immunofluorescence. Several high-quality antibodies that successfully detect PLC-gamma-2 under our standardized experimental conditions were identified. In our effort to address the antibody reliability and reproducibility challenges in scientific research, the authors recommend the antibodies that demonstrated to be underperforming under our standard procedure be removed from the commercial antibody market. However, the authors do not engage in result analysis or offer explicit antibody recommendations. A limitation of this study is the use of universal protocols - any conclusions remain relevant within the confines of the experimental setup and cell line used in this study. Our primary aim is to deliver top-tier data to the scientific community, grounded in Open Science principles. This empowers experts to interpret the characterization data independently, enabling them to make informed choices regarding the most suitable antibodies for their specific experimental needs. Guidelines on how to interpret the antibody characterization data in this study are openly available. ^29^

The underlying data can be found on Zenodo, an open-access repository for which YCharOS has its own collection of antibody characterization reports. ^30^^,^^31^

## Methods

### Antibodies

All PLC-gamma-2 antibodies are listed in Table 2, along with their corresponding Research Resource Identifiers, or RRID, to ensure the antibodies are cited properly. ^32^ Peroxidase-conjugated goat anti-rabbit and anti-mouse antibodies are from Thermo Fisher Scientific (cat. number 65-6120 and 62-6520). Alexa-555-conjugated goat anti-rabbit and anti-mouse secondary antibodies are from Thermo Fisher Scientific (cat. number A21429 and A21424).

**Table 2.** Summary of the PLC-gamma-2 antibodies tested.

Company	Catalog number	Lot number	RRID (Antibody Registry)	Clonality	Clone ID	Host	Concentration (μg/μL)	Vendors recommended applications
Abcam	ab109267 ** ^a^	GR498527	AB_10888119	recombinant-mono	EPR1403	rabbit	1.32	WB
Abcam	ab133522 **	GR962819	AB_2927390	recombinant-mono	EPR5914-34	rabbit	0.63	WB
R&D Systems (a Bio-Techne brand)	MAB3716 *	XXN0319021	AB_2163529	monoclonal	346404	mouse	0.50	WB, IF
Novus Biologicals (a Bio-Techne brand)	NBP2-52536 * ^a^	141225	AB_2927391	monoclonal	2D9E8	mouse	1.00	WB, IF
Cell Signaling Technology	3872	5	AB_2299586	polyclonal	-	rabbit	0.12	WB, IP
Cell Signaling Technology	34264 **	1	AB_2927393	recombinant-mono	E7L6G	rabbit	0.39	WB
Cell Signaling Technology	55512 **	1	AB_2799488	recombinant-mono	E5U4T	rabbit	0.05	WB, IP, IF
GeneTex	GTX111178	40058	AB_1951276	polyclonal	-	rabbit	0.66	WB, IF
GeneTex	GTX111293	43334	AB_1951274	polyclonal	-	rabbit	0.75	WB
Thermo Fisher Scientific	MA5-35670 **	XH3670294	AB_2849570	recombinant-mono	ARC1176	rabbit	0.73	WB
Thermo Fisher Scientific	MA5-38600 *	XH3669859	AB_2898512	monoclonal	2D9E8	mouse	1.00	WB, IF

WB=Western Blot; IF= immunofluorescence; IP=immunoprecipitation.

* Monoclonal antibody.

** Recombinant antibody.

^a^ Antibodies that have been discontinued following this study.

### Cell culture

Both THP-1 and *PLCG2* KO cell lines used are listed in Table 1, together with their corresponding RRID to ensure the cell lines are cited properly. ^33^ Cells were cultured in RPMI 1640 medium (Thermo Fisher Scientific, cat. number 11875119) containing 10% fetal bovine serum (Wisent, cat. number 080450), 2 mM L-glutamate (Wisent, cat. number 609065), 100 IU penicillin and 100 μg/mL streptomycin (Wisent, cat. number 450201).

One set of the THP-1 WT and *PLCG2* KO cells were treated with 200 ng/mL of PMA (Abcam, cat. number ab147465) for 2 days. 200 ng/mL of PMA was added to fresh medium on both day 1 and day 2. ^34^ The other set of cells, WT and *PLCG2* KO remained untreated.

### Antibody screening by Western Blot

Western Blots were performed as described in our standard operating procedure. ^27^^,^^28^ THP-1 WT and *PLCG2* KO were collected in RIPA buffer (25 mM Tris-HCl pH 7.6, 150 mM NaCl, 1% NP-40, 1% sodium deoxycholate, 0.1% SDS) supplemented with 1× protease inhibitor cocktail mix (MilliporeSigma, cat. number P8340). Lysates were sonicated briefly and incubated for 30 min on ice. Lysates were spun at ~110,000 × g for 15 min at 4°C and equal protein aliquots of the supernatants were analyzed by SDS-PAGE and Western Blot. BLUelf prestained protein ladder from GeneDireX (cat. number PM008-0500) was used.

Western Blots were performed with precast midi 4-20% Tris-Glycine polyacrylamide gels from Thermo Fisher Scientific (cat. number WXP42012BOX) ran with Tris/Glycine/SDS buffer from bio-Rad (cat. number 1610772), loaded in Laemmli loading sample buffer from Thermo Fisher Scientific (cat. number AAJ61337AD) and transferred on nitrocellulose membranes. Proteins on the blots were visualized with Ponceau S staining (Thermo Fisher Scientific, cat. number BP103-10) which is scanned to show together with individual Western Blot. Blots were blocked with 5% milk for 1 hr, and antibodies were incubated overnight at 4°C with 5% milk in TBS with 0.1% Tween 20 (TBST) (Cell Signalling Technology, cat. number 9997). Following three washes with TBST, the peroxidase conjugated secondary antibody was incubated at a dilution of ~0.2 μg/mL in TBST with 5% milk for 1 hr at room temperature followed by three washes with TBST. Membranes were incubated with Pierce ECL from Thermo Fisher Scientific (cat. number 32106) Or Clarity Western ECL Substrate from Bio-Rad (cat. number 1705061) prior to detection with the iBright™ CL1500 Imaging System from Thermo Fisher Scientific (cat. number A44240).

### Antibody screening by immunoprecipitation

Immunoprecipitation was performed as described in our standard operating procedure. ^27^^,^^28^ Antibody-bead conjugates were prepared by adding 10 μL of antibodies 3872 and 55512** or 2 μg of the remaining antibodies tested to 500 μL of Pierce IP Lysis Buffer from Thermo Fisher Scientific (cat. number 87788) in a 1.5 mL microcentrifuge tube, together with 30 μL of Dynabeads protein A - (for rabbit antibodies) or protein G - (for mouse antibodies) from Thermo Fisher Scientific (cat. number 10002D and 10004D, respectively). The low concentrations of antibodies 3872 and 55512** can account for why 10 μL was added to make the antibody-bead conjugates, rather than 2 μg. Tubes were rocked ~1 hr at 4°C followed by several washes to remove unbound antibodies.

THP-1 WT were collected in Pierce IP buffer (25 mM Tris-HCl pH 7.4, 150 mM NaCl, 1 mM EDTA, 1% NP-40 and 5% glycerol) supplemented with protease inhibitor. Lysates were rocked 30 min at 4°C and spun at 110,000 × g for 15 min at 4°C. 0.5 mL aliquots at 2.0 mg/mL of lysate were incubated with an antibody-bead conjugate for ~1 hr at 4°C. The unbound fractions were collected, and beads were subsequently washed three times with 1.0 mL of IP lysis buffer and processed for SDS-PAGE and Western Blot on a precast midi 4-20% Tris-Glycine polyacrylamide gels from Thermo Fisher Scientific.

### Antibody screening by immunofluorescence

Immunofluorescence was performed as described in our standard operating procedure. ^27^^,^^28^ THP-1 WT and *PLCG2* KO were labelled with a green and a far-red fluorescence dye, respectively. The fluorescent dyes used are from Thermo Fisher Scientific (cat. number C2925 and C34565). The nuclei were labelled with DAPI (Thermo Fisher Scientific, cat. number D3571) fluorescent stain. WT and KO cells were plated in 96-well plate with optically clear flat bottom (Perkin Elmer, cat. number 6055300) as a mosaic and incubated for 24 hrs in a cell culture incubator at 37 ^o^C, 5% CO _2_. Cells were fixed in 4% paraformaldehyde (PFA) (Beantown chemical, cat. number 140770-10 ml) in phosphate buffered saline (PBS) (Wisent, cat. number 311-010-CL) for 15 min at room temperature and then washed 3 times with PBS. Cells were permeabilized in PBS with 0.1% Triton X-100 (Thermo Fisher Scientific, cat. number BP151-500) for 10 min at room temperature and blocked with PBS with 5% bovine serum albumin (BSA) (Wisent, cat. number 800-095), 5% goat serum (Gibco, cat. number 16210-064) and 0.01% Triton X-100 for 30 min at room temperature. Cells were incubated with IF buffer (PBS, 5% BSA, 0.01% Triton X-100) containing the primary PLC-gamma-2 antibodies overnight at 4°C. Cells were then washed 3 × 10 min with IF buffer and incubated with corresponding Alexa Fluor 555-conjugated secondary antibodies in IF buffer at a dilution of 1.0 μg/mL for 1 hr at room temperature with DAPI. Cells were washed 3 × 10 min with IF buffer and once with PBS.

Images were acquired on an ImageXpress micro widefield high-content microscopy system (Molecular Devices), using a 20× NA 0.95 water objective lens and scientific CMOS camera (16-bit, 1.97 mm field of view), equipped with 395, 475, 555 and 635 nm solid state LED lights (Lumencor Aura III light engine) and bandpass emission filters (432/36 nm, 520/35 nm, 600/37 nm and 692/40 nm) to excite and capture fluorescence emission for DAPI, CellTrackerTM Green, Alexa fluor 555 and CellTrackerTM Red, respectively. Images had pixel sizes of 0.68 × 0.68 microns. Exposure time was set with maximal (relevant) pixel intensity ~80% of dynamic range and verified on multiple wells before acquisition. Since the IF staining varied depending on the primary antibody used, the exposure time was set using the most intensely stained well as reference. Frequently, the focal plane varied slightly within a single field of view. To remedy this issue, a stack of three images per channel was acquired at a z-interval of 4 microns per field and best focus projections were generated during the acquisition (MetaExpress v6.7.1, Molecular Devices). Segmentation was carried out on the projections of CellTrackerTM channels using CellPose ^35^ v1.0 on green (WT) and far-red (KO) channels, using as parameters the ‘cyto’ model to detect whole cells, and using an estimated diameter tested for each cell type, between 15 and 20 microns. Masks were used to generate cell outlines for intensity quantification. Figures were assembled with Adobe Photoshop (version 24.1.2) to adjust contrast then assembled with Adobe Illustrator (version 27.3.1).

## Data Availability

Zenodo: Antibody Characterization Report for PLC-gamma-2, https://doi.org/10.5281/zenodo.10108291. ^30^ Zenodo: Dataset for the PLC-gamma-2 antibody screening study, https://doi.org/10.5281/zenodo.10149969. ^31^ Data are available under the terms of the Creative Commons Attribution 4.0 International license (CC-BY 4.0).
