# Supplementary material for: A guide to selecting high-performing antibodies for PLC-gamma-2 for use in Western Blot, immunoprecipitation and immunofluorescence
Source: F1000Res. 2024 Jan 18;13:77. [Version 1] doi: 10.12688/f1000research.146156.1 (PMC11489847; doi:10.12688/f1000research.146156.1)
Supplement: Peer review history

# Reviewer report: Reviewer response for version 1

**DOI:** 10.5256/f1000research.160203.r330318

**Published:** 2024-10-18

**Authors:** Deborah Moshinsky

**Affiliations:** 1 Institute for Protein Innovation, Boston, Massachusetts, USA

The authors present a scientific basis for examining PLC-gamma-2 antibody performance and tested 11 commercially available antibodies against this target in Western Blot (WB), IP, and IF using standardized protocols.  WB, IP, and IF data for each of the 11 antibodies were shown, allowing readers to assess Ab performance.  The methods were clearly described and links to the underlying data were provided.  This publication should be helpful for researchers of PLC-gamma-2 to assess commercially available antibody performance and choose the most promising antibodies for validation or use in their application.

Are sufficient details of methods and materials provided to allow replication by others?

Yes

Is the rationale for creating the dataset(s) clearly described?

Yes

Are the datasets clearly presented in a useable and accessible format?

Yes

Are the protocols appropriate and is the work technically sound?

Yes

Reviewer Expertise:

Antibody Characterization and Validation

I confirm that I have read this submission and believe that I have an appropriate level of expertise to confirm that it is of an acceptable scientific standard.

# Reviewer report: Reviewer response for version 1

**DOI:** 10.5256/f1000research.160203.r300907

**Published:** 2024-08-05

**Authors:** Abhinay Ramaprasad

**Affiliations:** 1 The Francis Crick Institute, London, UK

This data note compares the performance of 11 commercially available antibodies for PLC-gamma-2 with the aim to act as a guide for readers to choose appropriate antibodies in their studies and to address antibody reproducibility issues. The authors have performed careful, standardised Western blot, immunoprecipitation and immunofluorescence experiments with wildtype and KO cells to draw comparisons. Whilst they abstain from making recommendations for good reasons, the results clearly show some antibodies outperforming others. The experiments are done well with appropriate controls and reported in clear and informative manner.

 I have only two minor suggestions that might further improve interpretation by readers-

 1) Could the authors explain the use of PMA for general readers not familiar with the field?

 2) The authors say "Quantification of immunofluorescence intensity in hundreds of WT and KO cells was performed for each antibody tested". However, I was unable to find this data. It would be helpful if this data is provided as a dotplot in Figure 3 along with the images which represent only few select cells and at times, hard to interpret.

Are sufficient details of methods and materials provided to allow replication by others?

Yes

Is the rationale for creating the dataset(s) clearly described?

Yes

Are the datasets clearly presented in a useable and accessible format?

Yes

Are the protocols appropriate and is the work technically sound?

Yes

Reviewer Expertise:

Biochemistry, Malaria, Conditional mutagenesis, phospholipases, protein function discovery.

I confirm that I have read this submission and believe that I have an appropriate level of expertise to confirm that it is of an acceptable scientific standard.
